# Recent Progress with In Situ Characterization of Interfacial Structures under a Solid–Gas Atmosphere by HP-STM and AP-XPS

**DOI:** 10.3390/ma12223674

**Published:** 2019-11-07

**Authors:** Huan Zhang, Haoliang Sun, Kongchao Shen, Jinping Hu, Jinbang Hu, Zheng Jiang, Fei Song

**Affiliations:** 1 Shanghai Institute of Applied Physics, Chinese Academy of Sciences, Shanghai 201800, China; zhanghuan@sinap.ac.cn (H.Z.); sunhaoliang@sinap.ac.cn (H.S.); 11636007@zju.edu.cn (K.S.); hujinping@sinap.ac.cn (J.H.); hujinbang@sinap.ac.cn (J.H.); jiangzheng@sinap.ac.cn (Z.J.); 2 University of Chinese Academy of Sciences, Beijing 101000, China; 3 Shanghai Synchrotron Radiation Facility, Zhangjiang Laboratory, Chinese Academy of Sciences, Shanghai 201204, China

**Keywords:** HP-STM, AP-XPS, interfacial structures, solid–gas atmosphere

## Abstract

Surface science is an interdisciplinary field involving various subjects such as physics, chemistry, materials, biology and so on, and it plays an increasingly momentous role in both fundamental research and industrial applications. Despite the encouraging progress in characterizing surface/interface nanostructures with atomic and orbital precision under ultra-high-vacuum (UHV) conditions, investigating in situ reactions/processes occurring at the surface/interface under operando conditions becomes a crucial challenge in the field of surface catalysis and surface electrochemistry. Promoted by such pressing demands, high-pressure scanning tunneling microscopy (HP-STM) and ambient pressure X-ray photoelectron spectroscopy (AP-XPS), for example, have been designed to conduct measurements under operando conditions on the basis of conventional scanning tunneling microscopy (STM) and photoemission spectroscopy, which are proving to become powerful techniques to study various heterogeneous catalytic reactions on the surface. This report reviews the development of HP-STM and AP-XPS facilities and the application of HP-STM and AP-XPS on fine investigations of heterogeneous catalytic reactions via evolutions of both surface morphology and electronic structures, including dehydrogenation, CO oxidation on metal-based substrates, and so on. In the end, a perspective is also given regarding the combination of in situ X-ray photoelectron spectroscopy (XPS) and STM towards the identification of the structure–performance relationship.

## 1. Introduction

With the rapid pace of characterization technologies in surface science during the past several decades, techniques with static investigation capabilities with atomic precision and orbital resolution have been emerging [1,2,3,4,5] in applications in such as materials science, energy conversion and storage, catalysis, solar cells, and so on. Specifically, numerous spectroscopes and microscopes have been developed, such as optical microscopy (from the very beginning), electron microscopy (nowadays), infrared spectroscopy (IR), X-ray diffraction (XRD), scanning force microscopy (SFM), scanning tunneling microscopy (STM), photoelectron spectroscopy (PES) (especially, X-ray photoelectron spectroscopy, XPS), and so on. In particular, STM and XPS have grown to be two of the most universal and convenient characterization techniques [6,7] for investigation fine structures in surface science.

It is well accepted that STM can provide atomically resolved information of nanostructures on the surface by measuring the tunneling current between a sharp metallic tip and a conducting/semiconducting sample, where the tunneling current *I* is calculated as:

(1) $$I=kVe^{-l\sqrt{\Phi }}S$$

where *k* and *l* are constant, *V* is the bias voltage applied between tip and sample, *S* is the distance between tip and sample, and *Φ* is the average work function of tip and sample, which is a fixed value for a certain sample and tip [8]. This was made possible thanks to Gerd Binnig and Heinrich Rohrer, who built the first STM at IBM Laboratory and won the Nobel prize in 1986 for this great invention. It has been encouraging for scientists to get atomic structures as well as the real-time information about surface morphology. According to the quantum tunneling theory, electrons are able to overcome a certain energy barrier and hop to a different position by a tunneling effect. Meanwhile, the tunneling current is produced when the distance between sample and tip is less than 1 nm with the help of a bias voltage. Nevertheless, the change of *S* results in the corresponding variation of tunneling current *I*. Consequently, surface morphology can then be directly obtained by measuring changes of the tunneling current. STM can work in either constant-current or constant-height modes, depending on the surface roughness. The most extensive application of STM is to manipulate atoms. Importantly, even tiny vibrations can significantly affect the stability of the STM instrument, which can inevitably affect the accuracy of STM results [9]. Therefore, vibration and noise reduction devices are generally coupled with the STM system [8]. In other words, STM is a technique which can easily obtain the atomic information of surface nanostructures and topology without the need of external light/beam or electrical/optical lens, and the STM investigation is nondestructive to samples. 

**Scheme 1:**
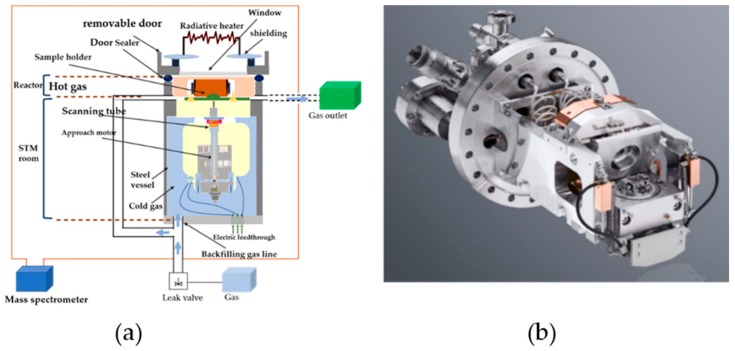
(**a**) Simulation diagram of the in-situ reaction cell of NAP-STM. (**b**) Picture of setup of NAP-STM.

At the beginning, STM experiments can only be operated either an under ultra-high-vacuum (UHV, below 10^−9^ mbar) environment or in air (10^3^ mbar) [10], whilst great success has been witnessed in revealing atomic structures and manipulating atoms on ideal crystal surfaces [11,12,13,14,15]. However, the UHV environment or in-air atmosphere does not match the reality of in situ reactions or operando conditions. Therefore, such a pressure gap needs to be bridged in order to investigate the changes and evolution of the surface morphology during reactions [9], and near-ambient pressure scanning tunneling microscopy (NAP-STM) (also called high-pressure scanning tunneling microscopy, HP-STM). As can be seen in Scheme 1, the heart of NAP-STM is the in-situ reaction cell [16], which holds the sample stage, heating facility, tip and scanning motors, and gas inlet system and is isolated from the main chamber. The reaction cell can be filled with reacting gas varying from 1 to 100 mbar during operando reactions, while the base pressure in the main chamber is maintained around 10^−7^ to 10^−9^ mbar depending on the differential pumping efficiency. By this means, any changes of nanostructures/defects/vacancies on the surface under operando conditions can be directly imaged, bridging the gap between model systems under the UHV environment and real catalytic reactions occurring in the field of chemical engineering [17].

**Scheme 2:**
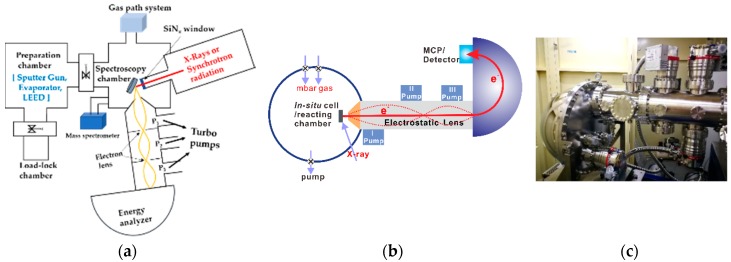
(**a**) Diagram of the setup of APXPS. (**b**)Principles of ambient pressure X-ray photoelectron spectroscopy (XPS) with differential pumping systems. (**c**) Picture of setup of APXPS.

With regard to XPS, a similar bottleneck has also been witnessed, since research from industry fields asks for in situ monitoring of changes to electronic structures at the surface/interface during reactions and not just the electronic structures or core level spectroscopy at certain steps. Meanwhile, there is a critical requirement that photoelectrons have to be in the UHV environment in order to be detected by the channel plate. Under such a background, the first ambient pressure X-ray photoelectron spectroscopy (AP-XPS) instrument was built by Kai Siegbahn’s group from Uppsala University [18]. As an advancement of UHV-XPS, near-ambient XPS adopts a special differential pumping system combined with electrostatic lens (as shown in Scheme 2a), keeping the electron analyzer still in the pressure range between 10^−9^ and 10^−10^ mbar, which can still work normally without being affected by the high-pressure environment in reaction chambers [19,20]. Consequently, measurements under relatively high pressures up to several dozens millibars [21,22,23,24,25,26] can be carried out without disturbing the electron analyzer, and more realistic results close to actual reactions can be obtained compared to UHV studies of ideal static surface/interfaces. 

Before stepping into the discussion of the AP-XPS application, we will first give a brief introduction of the differential pumping system (Scheme 2a). The front part is a sharp cone with a tiny hole (aperture) acting as the first differential pump. After getting through this hole, emitted photoelectrons have to pass another three differential pumping systems (usually pumped by turbos) before reaching the channel plate in the energy analyzer (Scheme 2b,c). Different hole sizes have great influences on the passing rate of photoelectrons [27]. The larger the hole size, the higher the electron passing rate. However, if the aperture is too large, it would break the high vacuum in the electron energy analyzer. On the other hand, if the aperture is too small, most photoelectrons are blocked outside of the cone, resulting in a very tiny signal in spectroscopy, although a relatively high pressure in the reaction chamber is guaranteed. Therefore, the size of the aperture is an important parameter for the maximum pressure. At present, the diameter of the aperture is generally chosen to be around 0.3–0.5 mm [28]. In addition, the collection efficiency of photoelectrons is greatly improved, as an extra electrostatic lens with a focus function to tune the own trajectories of photoelectrons is added in each differential pumping stage.

Thanks to the boosting improvement of AP-XPS, measurements can be operated nowadays up to hundreds millibars to simulate operando conditions as best as possible. In general, most AP-XPS systems have been equipped with other surface characterization tools such as low-energy electron diffraction (LEED), sputtering guns, in situ annealing treatments by either radiative heating or laser heating, various evaporation sources, and so on. With such means, samples can be properly cleaned or prepared before in situ measurements. While AP-XPS can not only work in gas environments, it can also measure the solid/liquid interface. In this review, we focused on AP-XPS investigations on the solid–gas interface in a gas environment.

## 2. Applications of HP-STM in Identifying Surface Catalysis Reactions

STM has come into use in many fields under a wide range of sample conditions (temperature, pressure, electric bias, reaction gas, and so on), which plays an important role in surface science research, and relevant research productions have emerged endlessly. In recent decades, HP-STM has gradually spread because of its advantages with (i) atomic resolution images of samples in a dynamic environment and (ii) vivid changes to samples with reaction conditions. In the following, we present examples of HP-STM experiments on solid/gas and solid/vapor interfaces about self-assembly and dehydrogenation processes.

### 2.1. HP-STM Experiments of Single-Crystal Reactions and Reactant Gas

Heterogeneous catalysis, as well as chemical, electrochemical, or physical interactions between the metal and the environmental medium, would cause reconstruction and aggregation on the surface, which is a very common subject in surface science. In this perspective, STM applications between single crystal and reactant gas are playing a central role for synthesizing efficient and stable catalysts in applications such as catalysis, energy, industry, corrosion, fuel cells, and so on.

#### 2.1.1. Reconstruction

**Figure 1:**
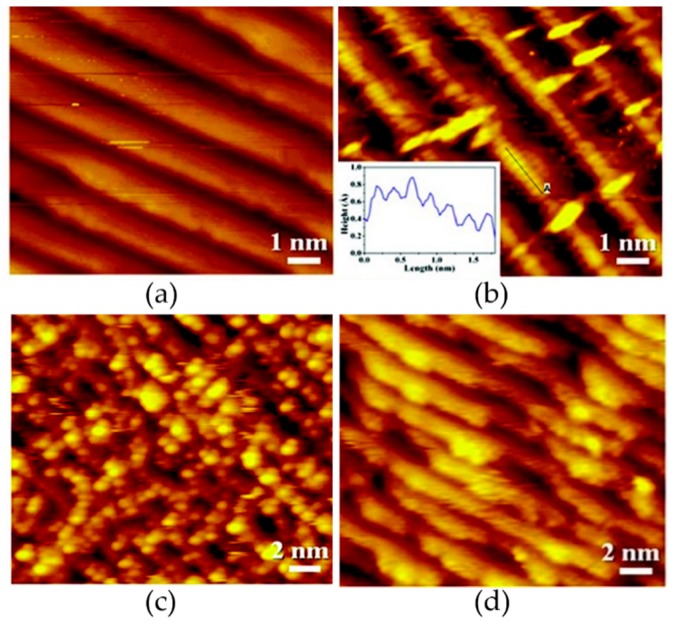
STM images of Pt(557) taken (**a**) under ultra-high-vacuum (UHV) conditions (8 × 10^−11^ Torr); (**b**) in 1 Torr of O_2_ for 30 min and (**c**) 2 h after continuous exposure of the clean Pt(557) to O_2_ gas; (**d**) after evacuating the high-performance (HP)-STM cell to 10^−8^ Torr for 1.5 h, it can be clearly seen that the clusters degenerate into distinct step edges.

With the change of gas content on the metal crystal surfaces, the restructuring process on crystal surfaces produces different effects. Zhu et al. observed nanometer-sized clusters after exposure to oxygen, which were detected by STM distinctly, as shown in Figure 1, and identified as Pt surface oxides by AP-XPS [29]. The nanometer-sized clusters gathered on Pt(557) at 1 Torr of O_2_ at room temperature (RT) (Figure 1b). These clusters firstly manifested as 1D chain structures at the step edges. With the gradual increase of exposure time, the chains progressively formed nanometer-sized clusters centered on step sites (Figure 1c). At last, the clusters disappeared on the crystal surface after evacuating O_2_ to UHV (Figure 1d). The experiment is highly repeatable, which ensures the accuracy and authenticity of the experiment results [29]. Besides, surface reconstruction can be promoted by adsorption and chemical reactions. Unsaturated sites with different contents facilitate surface cluster formations and step coalescence as a result of molecule adsorption. Liu et al. found a reconstruction of a 1 × 1 structure to a 2 × 1 structure on the ZnO ($10\bar{1}0$) surface under the introduction of H_2_ [30], resulting from the hydroxylation of the surface. Previous studies have shown that H_2_O adsorption can also lead to hydroxylation of the ZnO ($10\bar{1}0$) surface, and STM studies have proven that 2 × 1 reconstruction occurred at the same time due to the alternating distribution of H_2_O molecular and dissociation states [31]. According to comparative experiments and analyses, authors ruled out that the same experimental results were not caused by trace amounts of H_2_O in H_2_. Instead, they considered that the dissociation and adsorption of H_2_ was capable of contributing to the reconstruction of ZnO ($10\bar{1}0$). Consequently, we can conclude that many factors exist in metal reconstruction.

**Figure 2:**
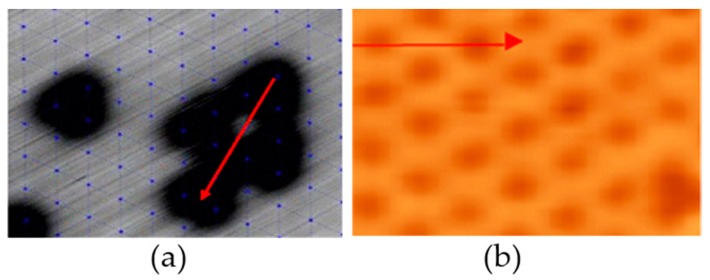
STM images for O adsorbed on Pd (111) forming a 2 × 2 superstructure. (**a**) Measured with a Pt tip, prepared by thermal dissociation of a layer of adsorbed molecules from 60 to 160 K. The underlying metal surface is also shown. (**b**) Measured with an Ir tip, prepared by exposure to oxygen gas at room temperature.

Interestingly, the same O/Pd (111)-2 × 2 system characterized by STM may present different images, as seen in Blanco et al. A Pd (111) surface with low coverage of oxygen atoms is presented in Figure 2a, which is deposited by thermal dissociation of a layer of adsorbed molecules from 60 to 160 K, inducing 2 × 2 ordering islands [32]. As a comparison, the Pd (111) surface is covered by a monolayer of oxygen atoms while being exposed to O_2_ at room temperature, as shown in Figure 2b. Another difference between the two sets of experiments is that a Pt tip under a low bias (*V* = 0.059 V and *I* = 16.9 nA) is used in the first system, while an Ir tip under a high bias voltage (*V* = 1.4 V and *I* = 0.7 nA) is used in the second one. Compared with Figure 2b, the substrate Pd can be clearly seen in Figure 2a. The oxygen atoms adsorbed on hollow fcc sites formed a triangle on the Pd surface and produced a depression. In Figure 2b, the oxygen atoms are imaged as protrusions, which spread over the entire Pd surface in the form of connected circles. Blanco et al. considered that the corrugation along the relevant scan line matters, it also has been predicted that the STM images rely heavily on the relative corrugation heights of the top or hcp positions along the scan line according to the calculations and experimental data [6]. In consequence, the main reason that leads to the difference between Figure 2a,b is the different corrugation heights of the top position and that of the hcp sites by using different tips and voltages. When the corrugation height of the top position is higher than that of the hcp site, a triangular shape is obtained. When it comes to the same corrugation height, the shape of the oxygen depression becomes circular. When the corrugation height of the top position is lower than that of the hcp site, the triangular shape reappears but is inverted relative to the one of Figure 2a.

**Figure 3:**
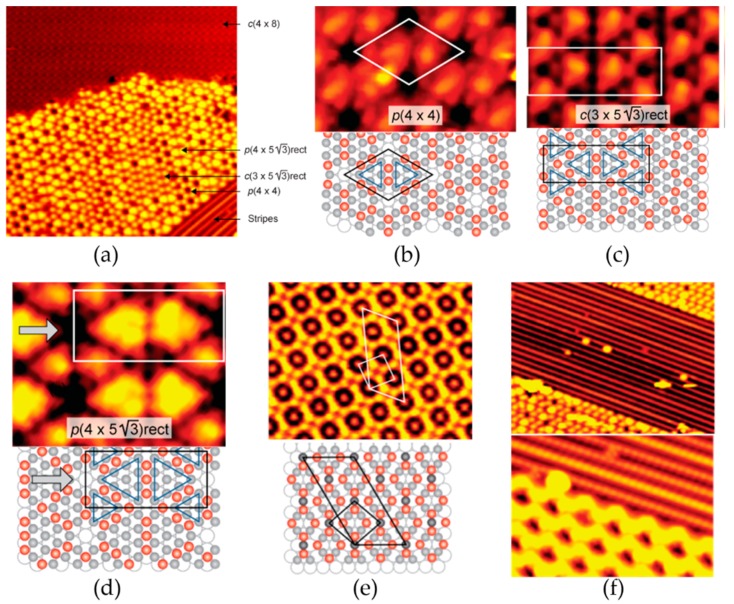
(**a**) STM image of an Ag (111) sample exposed to 5 × 10^−8^ Torr atomic oxygen for 40 min at 500 K sample temperature. STM images and experimentally derived structures of (**b**) p (4 × 4) phase, (**c**) c (3 × 5$\sqrt{3}$)rect, (**d**) p (4 × 5$\sqrt{3}$)rect, and (**e**) c (4 × 8) phase. Red/dark gray spheres represent the oxygen/second layer silver, (medium) gray represent the overlayer silver, and the open spheres represent the Ag (111) substrate atoms. (**f**) 200 × 200 Å and 75 × 75 Å for STM images of the stripe phase.

It has been a long time since single-crystal studies were carried out in the sixties and early seventies. Studies from the early years have been overthrown and revised by conclusions drawn by emerging advanced characterization tools. There are mainly the following stages for the understanding of Ag/O systems: (a) the p (4 × 4) phase, (b) the p (4 × 4) phase and an atomic adsorbate layer (including a phase with a local p ($\sqrt{3}$ × $\sqrt{3}$) R30° symmetry), and (c) the existence of phases containing both less than, equally as much, and more oxygen than the p (4 × 4) phase [33,34,35], with the help of combination of STM, surface x-ray diffraction (SXRD), XPS and density functional theory (DFT) calculations [35]. Schnadt et al. found that further, newly unobserved phases may play an indispensable role in the Ag/O system and its catalytic progress. As shown in Figure 3a, it can be seen the coexistence of the p (4 × 4) phase, c (3 × 5$\sqrt{3}$)rect, p (4 × 5$\sqrt{3}$)rect, c (4 × 8), and “stripe”. The hole structure of the p (4 × 4) phase is indicated in Figure 3a, which is consistent with previously reported characteristics. The hole structure has a positive impact on catalytic performance, which could hold and absorb foreign atoms and molecules. In terms of the previous literature, the p (4 × 4) phase and c (3 × 5$\sqrt{3}$)rect consist of Ag_6_ building blocks with a Ag atom located on either hcp or fcc sites, respectively [36]. There exists one fcc triangle and one hcp triangle in each primitive unit cell of a dimer. The site differences between these two triangles bring about a slight asymmetry of the dimers (Figure 3b). The p (4 × 5$\sqrt{3}$)rect phases feel like an extension of the p (4 × 4) phase and c (3 × 5$\sqrt{3}$)rect, with voids between four Ag_10_ and two Ag_6_ triangles being c (3 × 5$\sqrt{3}$)rect-like and those between four Ag_6_ and two Ag_10_ being p (4 × 4)-like. Based on DFT calculations and STM stimulations, STM images and experimentally derived structures of the p (4 × 4) phase, c (3 × 5$\sqrt{3}$)rect, and p (4 × 5$\sqrt{3}$)rect are shown in Figure 3b–d respectively. The c (4 × 8) and stripe phases are newly discovered phases shown in Figure 3e,f. In the model of the c (4 × 8) phase (Figure 3e), the bright features are considered as Ag atoms, for oxygen atoms are invisible in STM images typically. The hypothetical structure of the Ag atoms in the c (4 × 8) structure is depicted in Figure 3e, in which Ag atoms coordinate with a lower silver rather than the fully Ag-coordinated Ag atoms in the centers of the triangles. This is illustrated by the much smaller height of the Ag atoms than the brightest features of the p (4 × 4) phase, which indicates that the oxidation degree of the c (4 × 8) phase structure is more saturated than other building block structures.

As for the stripe phase, it is assumed that the protruding features are composed of pure silver atoms in the first place. This possibility is ruled out for the following deduction: along the {$1\bar{1}0$} directions, the distance between each nearest feature is measured to be *b* = 2.89 Å, which corresponds to the Ag (111) nearest-neighbor distance. The expected distance of the rows in the perpendicular direction is $\sqrt{3}$*b*, and the width of a repetitive unit containing four stripes is $4\sqrt{3}$*b*. The former does not agree with the STM results, and the latter is inconsistent with the situation of placing all the silver atoms in the same lattice site. However, XPS data were also uncertain in ascertaining absolutely the coordination of oxygen atoms because the XPS data represents fully covered surfaces to some extent. It is worth mentioning that similar stripe structures with quite a high oxygen content have been observed on other metal surfaces, such as Pt (110) [37]. 

#### 2.1.2. CO Catalytic Oxidation

In recent years, studies on CO catalytic oxidation have been developed to address energy issues in chemical and petrochemical industries [38]. Many raw materials have not been rationally utilized because of their low efficiency [39]. The typical reaction of CO catalytic oxidation is often used as a model reaction to study the catalytic behavior of catalysts for complete oxidation profoundly because of its simplicity and feasibility of simulation. CO oxidation on metal/oxide-based heterogeneous catalysts is the most representative reaction to explore the mechanism of water−gas shift reaction [39,40]. On the one hand, Au, Pt, Pd, and other metals have the advantages of being highly catalytic for CO. On the other hand, an oxide such as TiO_2_ itself has the advantages of a large specific surface area and high porosity, which enables the metal particles to be evenly distributed on the support surface to reduce the possibility of agglomeration of metal catalysts, thus improving the catalytic activity of metal catalysts for CO oxidation [41,42,43,44,45,46].

As mentioned above, Pt, Pd, and so on, have good catalytic activities for CO catalysis; hence, a large number of experiments have been carried out based on these materials [41,42,47,48]. By applying a high-pressure, high-temperature scanning tunneling microscope, Hendriksen et al. discussed the catalytic oxidation of CO on Pd (100) and Pt (110) surfaces in a flow reactor switching from a CO-rich to O_2_-rich atmosphere [49,50]. An adsorbate-covered metallic surface and an oxide were observed on both surfaces. According to the experimental results, an obvious, high catalytic efficiency was found in oxide states. Meanwhile, surface roughness increased step-by-step with the P_O2_:P_CO_ ratios. Similar results were also concluded by Li et al. [51]. The authors respectively covered the nano-thickness of SiO_2_ films on the polished front surface and unpolished back surface of the Pt(111) single crystal, and then they tested for CO oxidation activity at different temperatures. The reaction rates of CO oxidation on Pt(111) without SiO_2_ coverage, Pt(111) with SiO_2_ covered on the back side, and Pt(111) with SiO_2_ covered on both front and back were measured. Experimental data showed that the CO catalytic activity of the unpolished surface was absolutely higher than that of the polished surface, which may be due to the fact that atoms on the polished front side are equivalent to each other and equivalent to the same coordination number of 9. While on the unpolished back surface, atoms are mutually not equivalent, which gives rise to many more active sites.

There are also many studies of CO oxidation on Au/oxide systems by STM. Peters et al. exposed an Au (111) surface to CO gas with pressure varied from 0.1 to 530 mbar at 300 K, which caused surface disorder. The disorder remained until the evacuation of CO. The authors believed that irreversible, dissociative adsorption must have occurred [52]. Bertolini and his co-workers carried out similar experiments, in which Au (110) was exposed at CO pressures above 0.1 Torr [53]. An obvious surface roughness was found. The CO adsorption decreased unexpectedly with the increase of CO exposure time. It is considered that CO dissociates on Au substrates accompanied with carbon deposition. Peters et al. also reached similar conclusions: the morphological changes of Au (110) surfaces were irreversible after CO evacuation at room temperature [54]. As for the Au/oxide, Starr et al. observed the morphology of Au particles supported on thin FeO (111) films at CO pressures of 10^−6^, 10^−4^, 10^−3^ and 2 mbar; O_2_ pressure of 10^−5^ and 2 mbar; CO + O_2_ (1:1) pressures of 10^−6^, 10^−3^ and 2 mbar; and H_2_ pressures of 10^−5^ and 2 mbar at room temperature by in situ STM, respectively [55]. Au particles appear to be quite stable in O_2_ and H_2_ environments at pressures up to 2 mbar, while mobile Au species come into being for the destabilization of Au particles located at the step edges in CO and CO + O_2_ atmospheres. The Au particles in the latter case mainly locate at the step edges. At the same time, steps decorated by some ill-defined features cannot be observed on a FeO (111) thin film under CO without purification, but not under pure CO by comparing AES (Auger electron spectroscopy) data. Hence, Starr et al. considered that the reason for the phenomenon above is not the CO dissociation on the surface of Au particles at elevated pressures, but the impurity in CO-driven morphological changes.

### 2.2. HP-STM Experiments of Dehydrogenation Reactions

Dehydrogenation is the oxidation reaction of organic compounds in the presence of catalysts (chromium oxide, iron oxide, etc.) or dehydrogenation agents (sulfur, selenium, etc.) at high temperatures. It has been widely applied in organic synthesis, in which one of the hottest topics is about graphene. The dehydrogenation reactions on organic molecules provide many interesting inspirations for nanoribbon and graphene preparation. STM has played a more and more important and popular role in unraveling bond-breaking and bond-forming mechanisms of each step [56]. In general, catalytic dehydrogenation reactions aim to break carbon–hydrogen bonds of organic compounds to achieve the purpose of dehydrogenation while maintaining vulnerable carbon–carbon bonds, which consequently requires a suitable catalyst. On the other hand, oxygen is always added into the system in the oxidative dehydrogenation process. Oxygen makes it easier for H atoms to detach from X−H (X = N, C, O, ···) bonds and form H_2_O molecules, which is mainly used in the case where the organic compounds and its dehydrogenation products do not react with H_2_O. Thermal dehydrogenation raises the temperature of reactants, which could be seen in many reports. Sanchez-Sanchez et al. found that (cyclo)dehydrogenation reactions for C_60_H_30_ molecules on the TiO_2_ (110) surface are temperature-assisted [57]. Weak interactions between C_60_H_30_ molecules and the substrate TiO_2_ (110) surface exist at room temperature, as previously reported. Slight annealing deforms the interactions, quenches surface diffusion, and transforms diffuse organic molecules into chemisorbed molecules. Partial dehydrogenation occurs when the temperature continues to sire, and complete dehydrogenation occurs at 750 K. Sanchez-Sanchez et al. observed subsequently a bottom-up formation of assembled nanostructures on the dielectric surface in the way of the fully dehydrogenated molecules linking in a bottom-up coupling configuration. Gaudioso et al. proved that an extra voltage pulse (1.1–1.5 V) could also induce ethylene dehydrogenation on Ni (110) [58]. They identified dehydrogenated products by STM-IETS (inelastic electron tunneling spectroscopy) as two distinct types of acetylenes, S- and L-acetylene. Dehydrogenation products (acetylenes) would transform into further carbon atoms under a higher voltage (1.0–4.8 V).

Different reactants may also create the same dehydrogenation products. In the work of Montano et al., the same adsorbed structure, which was identified as the partially dehydrogenated π-allyl (C_6_H_9_), could be generated by exposing Pt(111) to the presence of 2 × 10^−6^ Torr cyclohexene or cyclohexane at 300 K [59]. The authors also conducted similar experiments under a mixture of 20 mTorr of H_2_ and 20 mTorr of cyclohexene at 300 K. They got a periodic π-allyl structure, which was similar to that obtained under pure cyclohexene conditions. The above indicates that the presence of 20 mTorr hydrogen is insufficient to influence the formation of the partially dehydrogenated π-allyl. Upon annealing to 350 K, periodic structures disappear, surfaces become disordered, and the expected cyclohexane and benzene are not detected in the gas phase composition by mass spectrometry. According to previous studies, the production of benzene rises as the temperature increases, but the desorption rate and mobility of benzene are lower than cyclohexane. It is considered that the surface is covered with benzene products with a self-poisoning and possibly immobile, carbonaceous overlayer because of the decomposition and dehydrogenation of adsorbed benzene at higher temperatures.

Another two comparative experiments were also carried out. One was based on the above experimental conditions with the extra introduction of 5 mTorr CO. At this time, the surface showed an ordered structure similar to the reported ($\sqrt{19}$ × $\sqrt{19}$) R23.4°-13CO structure [60,61]. It was concluded that CO displaced the original adsorbates on the surface, as seen through STM images with molecular resolution. The other experiment was repeated at 353 K. A disordered surface was observed again by STM, in which surface species were highly mobile. On the other hand, dehydrogenation is more likely to occur at higher temperatures, but abundant CO vacancies would be created on the sample to provide molecules with mobility at the same time [62,63]. However, surface became flat again when the sample was cooled down to 325 K. Therefore, Montano et al. proposed that the catalytic activity of Pt(111) was closely related to the mobility of surface molecules [59].

## 3. The Combination of HP-STM and AP-XPS

### 3.1. Investigations of Copper-Based Catalyst Reactions

As mentioned above, various CO catalytic oxidation reactions have important applications in many fields. Copper-based catalysts have been widely used in heterogeneous catalytic systems, such as water–gas shift and reverse water–gas shift$\;\left(\mathrm{CO}+H_{2}O\to {\mathrm{CO}}_{2}+H_{2}\right),$ partial oxidation $\left(2{\mathrm{CH}}_{3}\mathrm{OH}+O_{2}\to 2{\mathrm{CH}}_{2}O+2H_{2}O\;\mathrm{and}\;2{\mathrm{CH}}_{3}\mathrm{OH}+O_{2}\to 2{\mathrm{CO}}_{2}+4H_{2}\right)$, steam reforming $\left({\mathrm{CH}}_{3}\mathrm{OH}+H_{2}O\to {\mathrm{CO}}_{2}+3H_{2}\right)$, and, most typically, CO oxidation $\left(2\mathrm{CO}+O_{2}\to 2{\mathrm{CO}}_{2}\right)$. 

**Figure 4:**
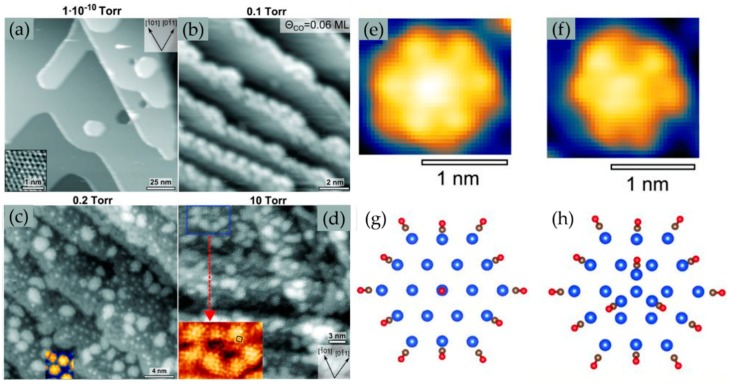
STM images of Cu(111) showing clusters filling the terraces as a function of ambient CO pressure. (**a**) In 1×10^−10^ Torr. (Bottom inset) Atomically resolved image. (**b**) Under 0.1 Torr of CO, clusters form at step edges. (**c**) Under 0.2 Torr of CO, clusters form on the terraces. (**d**) Under 10 Torr of CO, a high density of clusters with adsorbed CO molecules (expanded in the inset) completely covers the surface. (**e**,**f**) are enlarged images of clusters with C6 symmetry or C_3_ symmetry. (**g**,**h**) are density functional theory (DFT)-optimized cluster models of (**e**,**f**).

With the combination of HP-STM and AP-XPS, surface structure information, including atomic resolution, chemical sensitivity, and valence changes, of Cu-based substrates can be investigated in situ in the reaction process, so as to have a comprehensive understanding of the reaction mechanisms of such heterogeneous catalytic reactions. Obvious changes in clusters were observed in the terraces of Cu(111) as a function of ambient CO pressure [64]. Micrometer-scale terraces and atomically resolved images were visible under UHV by STM (Figure 4a). A new structure appeared along the step edges after introduction of 0.1 Torr of CO (Figure 4b), and the terraces were gradually covered by nanoclusters after introduction of 0.2 Torr of CO (Figure 4c). Then, the density of clusters increased sharply under 10 Torr of CO (Figure 4d). After careful analysis of the structures in Figure 4c, the small clusters were ~0.5 nm in diameter, with poorly resolved triangular shapes, and larger ones were ~1.5 nm in diameter with hexagonal shapes. These two types of clusters are magnified in Figure 4e,f. It can be confirmed that the clusters were not composed of CO molecules, which are only a few angstroms high, and the height of clusters were close to that of the steps. These clusters kept moving as a result of thermal mobility. Time-lapse images by STM showed that Cu clusters formed by the splitting of Cu atoms from step edges followed by coalescence and accretion. Moreover, with the help of DFT calculations, the small clusters were assigned to three Cu atoms with an apparent height of about half that of a monatomic step (Figure 4g), the larger ones were considered as 19 Cu atoms with the height of about a monatomic step, whereas these Cu atoms took the shape of hexagonal, closed-shell structures with the hexagonal (C_6_) symmetry. The inferred structure with increased adsorption energy of CO at low-coordinated Cu atoms, and the lowering of the binding energy of metal atoms bound to CO, was proven to be the most stable configuration. In each case, the attachment of a CO molecule to each outermost Cu atom is necessary for the energetic stability of the systems, which is predicted by DFT calculations. Meanwhile, standard Bardeen approximation revealed that the CO molecules on the corners indeed had greater tunneling contributions than those on the edges, contributing to a higher brightness, which agreed well with other experiments. The formation of another cluster with C_3_ symmetry is also explained by following reason: three low-coordinated Cu atoms were added at the center of the 19-Cu-atom shell structure (Figure 4f,h), and these three Cu atoms attracted three additional CO molecules, which distorted the tilt angles of the peripheral CO molecules, and was consistent with experimental observations (Figure 4f).

**Figure 5:**
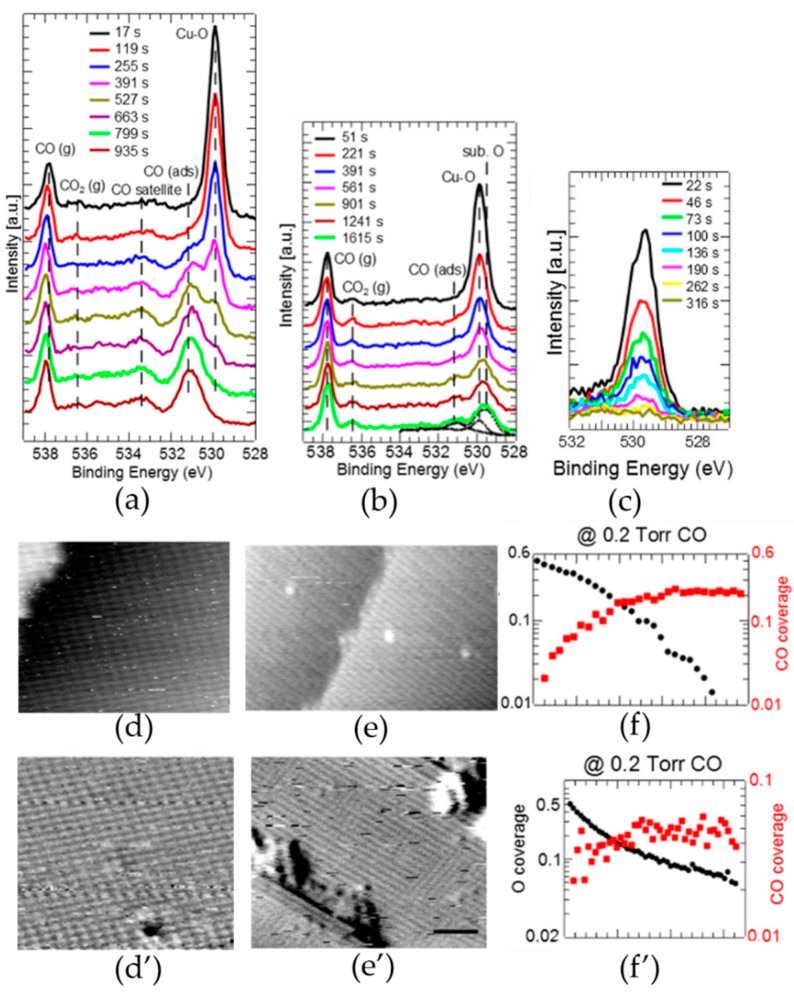
XPS O 1s spectral region of (**a**) the Cu(110)−(2 × 1)-O surface, (**b**) the Cu(100)−(2$\sqrt{2\;}\;\times \;\sqrt{2\;}$R45°)-O surface, (**c**) a surface oxide layer on Cu(111) under 0.2 Torr of CO at RT, indicating the presence of chemisorbed O at 529.9 eV, molecularly adsorbed CO at 531.0 eV, and gas-phase CO at 538 eV. STM images of the Cu(110)−(2 × 1)-O surface at RT (**d**) in UHV and (**d’**) under 0.01 Torr CO pressure after about 1 h following CO introduction, the Cu(100)−(2$\sqrt{2}\;\times \;\sqrt{2}$R45°)-O surface at RT (**e**) in UHV and (**e’**) under 0.01 Torr of CO after 0.5 h following CO introduction. Evolution of the adsorbed oxygen and CO coverage obtained from the area of Gaussian−Lorentzian fits to the XPS peaks under 0.2 Torr CO at RT for (**f**) the Cu(110)−(2 × 1)-O surface, (**f’**) the Cu(100)-(2$\sqrt{2\;}\;\times \;\sqrt{2\;}$R45°)-O surface.

Eren et al. also carried out several studies of CO oxidation on low-index Cu surfaces with a pre-adsorbed oxygen layer, in contrast to experiments under UHV [65]. As can be seen in Figure 5a–c, there were two O-containing species: a peak of chemisorbed O at 529.9 eV and another of molecularly adsorbed CO at 531.0 eV that changed over the reaction time. The intensity of the adsorbed CO peaks became gentler, and that of the chemisorbed oxygen peaks became sharper. It can be inferred that the chemisorbed oxygen was gradually replaced by the adsorbed CO during the reaction process. As proposed in previous literature, oxygen atoms on Cu(110) form 2 × 1 structures, mentioned as an “added-row structure” with 0.5 monolayer coverage. It produces 2$\sqrt{2}$ × $\sqrt{2}$R45° (“missing-row”) structures on Cu(100) with 0.5 monolayer (ML) coverage. STM images of the Cu(110)-(2 × 1)-O surface and the Cu(100)-(2$\sqrt{2}$ × $\sqrt{2}\;$R45°)-O surface at RT in UHV and under 0.01 Torr CO pressure for about 1 h after CO introduction are shown in Figure 5d,d’,e,e’. With the increase of CO pressure to 0.2 Torr, the O-removal rate increased over time, while the 2 × 1 structures on the oxygen-covered regions of the surface remained unchanged according to STM and LEED (low-energy electron diffraction). Similar trends were found on the Cu(100)-(2$\sqrt{2}\;$× $\sqrt{2}\;$R45°)-O surface, which were still visible under 0.01 Torr of CO up to 1 h, indicating the reaction rate of CO + O_2_ was slow (Figure 5e,e’). The functional relationships between the absorbed oxygen and CO coverage on both surfaces converted from XPS peaks after introduction of 0.2 Torr CO and time are shown in Figure 5f,f’. The intensities of XPS peaks of O and CO are plotted as a function of time and pressure. The reaction rates of CO on the above three substrates are inferred as Cu(111) > Cu(100) > Cu(110).

**Figure 6:**
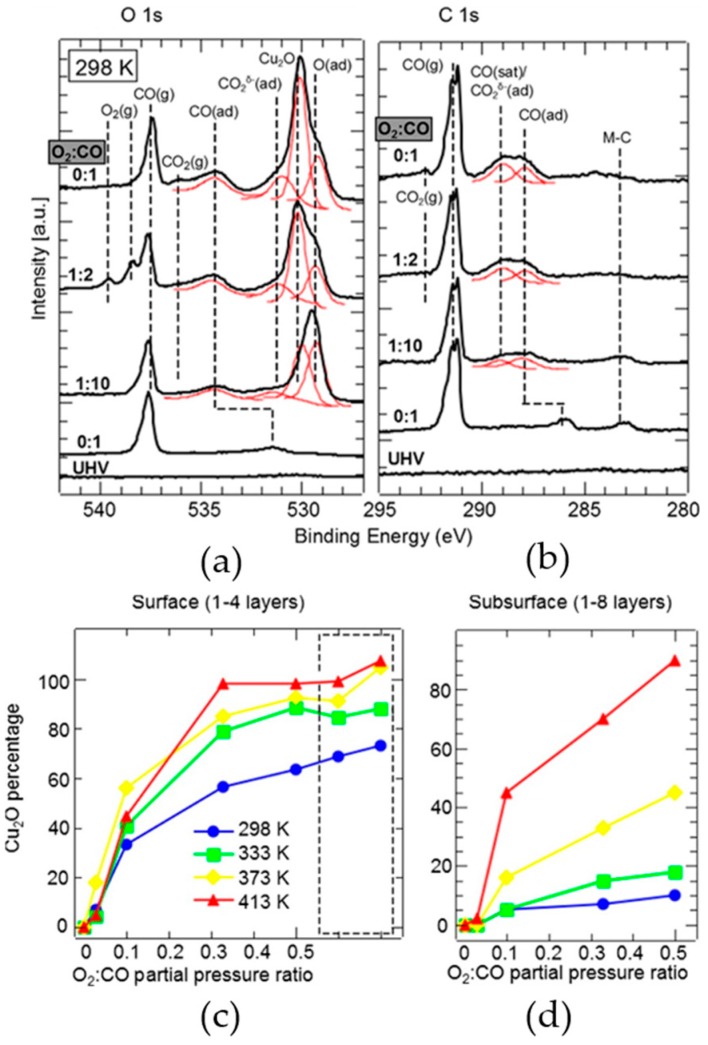
(**a**,**b**) are AP-XPS data of O 1s and C 1s peaks from a Cu(111) sample at 298 K. From bottom to top: in UHV, under 0.3 Torr of CO, under 0.03 Torr of O_2_ + 0.3 Torr of CO, under 0.15 Torr of O_2_ + 0.3 Torr of CO and after pumping the O_2_. Molecularly adsorbed CO peaks are observed at 531.5 eV of O 1s and 286.1 eV of C 1s on metallic Cu, and around 534.2 eV of O 1s, 287.9 eV of C 1s, and 289 eV (satellite) on the Cu_2_O covered region. The chemisorbed O peak is considered at 529.4 eV both on the metallic Cu and on Cu_2_O. The lattice O from Cu_2_O appears at 530.2 eV. An intermediate of the CO oxidation reaction, adsorbed CO_2_^δ-^, occurs at 531.5 eV of O 1s and 289 eV of C 1s. (**c**) Cu_2_O percentage (the rest is metallic Cu) of the surface estimated from the intensity ratio between O 1s (530.2 eV) and Cu 2p XPS peaks. (**d**) Cu_2_O percentage (the rest is metallic Cu) on the near-surface region (IMFP ~1.7 nm) estimated from the NEXAFS resonance at the Cu L3 edge.

Besides, Eren et al. showed the specialized chemical structure changes of a Cu(111) model catalyst during CO oxidation under various gas O_2_:CO ratios varied from 298 to 413 K by AP-XPS, as shown in Figure 6a,b [66]. As previously reported, the peak at 529.4 eV corresponds to the chemisorbed oxygen on the metallic Cu(111) surface [65,67,68]. The peak at 530.2 eV is considered as the O 1s peak of the Cu_2_O phase because the Cu 2p peaks of the CuO phase are typically 1.4 eV higher than those of Cu and Cu_2_O, which should have resulted in shoulder or satellite peaks at higher binding energies in spectroscopy results. The full width at half maximum and asymmetry parameter of the Sonjac–Dunjic functions of Cu 2p peaks remain unchanged during CO oxidation under various pressures and temperatures. Meanwhile, the possibility of subsurface O at 529.4 eV can be ruled out because the intensity ratio between this peak and that at 530.2 eV is significantly lower at *E*_ph_ = 1150 eV than at *E*_ph_ = 735 eV. The previous assignments are confirmed in AP-XPS experiments by introducing O_2_ into pure Cu_2_O. The assignments can also be confirmed by a significant peak growth of 529.4 eV and the absence of CuO in experimental results. With the increase of oxygen ratio, the thin Cu_2_O layer gradually covers the whole surface. Meanwhile, the peak of 529.4 eV still exists at this time, which is attributed to the chemisorbed O on metallic Cu or O adsorbed on defect sites of Cu_2_O.

Based on previous literature, the peaks at around 531.5 and 286.1 eV are assigned to O 1s and C 1s of CO adsorbed on metallic Cu(111). The peaks at around 531.4 and 288.4 eV are assigned to O 1s and C 1s of the reaction intermediate (CO_2_^δ–^) on Cu, while the data of those on Cu_2_O are absent. In their work, the peaks at around 531.5 and 289.0 eV are ascribed to CO2^δ–^ on Cu_2_O, and the peaks at around 534.2 and 287.9 eV are ascribed to CO on Cu_2_O. AP-XPS contrast experiments, in which pure Cu_2_O samples are placed under 0.1 Torr of CO and under 0.4 Torr of CO_2_ at RT, were carried out to confirm previous assignments. Results are exactly as conjectured, except that CO adsorption on Cu_2_O also results in an intense satellite peak at 289 eV, similar to those of CO adsorbed on metallic Cu.

The functional Cu_2_O percentages of the surface converted from the O 1s and Cu 2p XPS peak intensity ratios and O_2_:CO pressure ratio are shown in Figure 6c. Cu_2_O increases with the temperature. Above 333 K, more than 80% of the surface is oxidized to Cu_2_O when the ratio of O_2_: CO is higher than 0.3. After pumping O_2_ out of the chamber, and only CO is left, no significant reduction from Cu_2_O to Cu is observed at least for more than 0.5 h when T ≤ 413 K. Functional O_2_:CO pressure ratios and Cu_2_O percentage on the near-surface region (IMFP ~1.7 nm) converted from near edge X-ray absorption fine structure (NEXAFS) data at the Cu L3 edge is shown in Figure 6d. It can be seen that there is more improvement in the depth of information than that in Figure 6c, but the general trend is still the same as that in Figure 6c [66].

**Figure 7:**
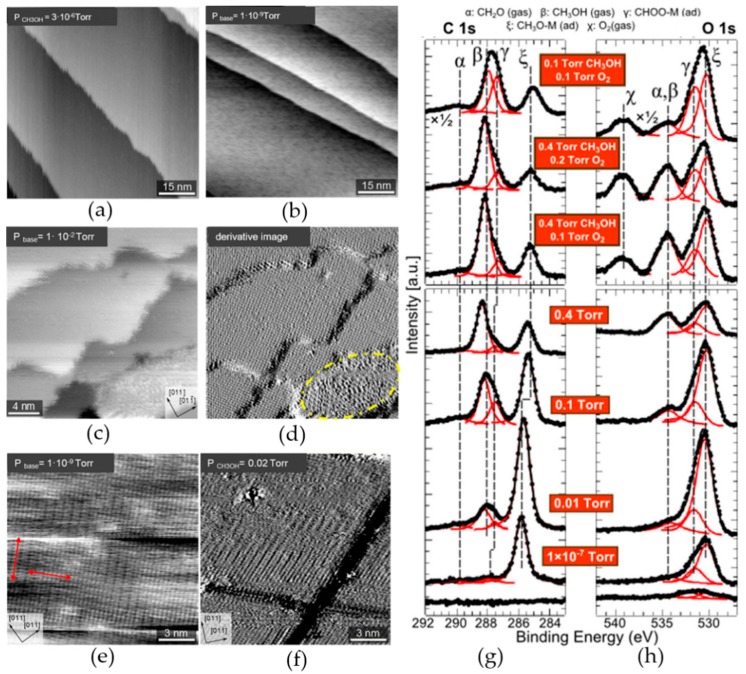
STM images of the Cu(100) surface at 298 K after preparation (**a**) at pressure of 1 × 10^−9^ Torr (mostly water in the background), (**b**) in the presence of 3 × 10^−6^ Torr of gas phase methanol. STM images of the Cu(100) surface at 298 K (**c**) in the presence of 0.01 Torr of gas phase methanol, (**d**) the derivative image of (**c**) to enhance the STM contrast on the terraces. The region enclosed by the yellow broken line has not yet been determined. STM images of the Cu(100)-(2$\sqrt{2}\;\times \;\sqrt{2}\;$R45°)-O surface at 298 K (**e**) at UHV, (**f**) in the presence of 0.02 Torr of CH_3_OH. (**g**) C 1s and (**h**) O 1s regions of the AP-XPS spectra of Cu(100) at 298 K in the presence of gas phase methanol (lower panels) and methanol mixed with O2 (upper panels). From bottom to top: in UHV, under 1 × 10^−7^ Torr of CH_3_OH, under 0.01 Torr of CH_3_OH, under 0.1 Torr of CH_3_OH, under 0.4 Torr of CH_3_OH, under 0.4 Torr of CH_3_OH + 0.1 Torr of O_2_, and under 0.4 Torr of CH_3_OH + 0.2 Torr of O_2_.

As mentioned in previous literature, reconstruction appears on the Cu(100) surface under O_2_, CO, and CO_2_ pressure [64,65,66]. Methanol is incapable of inducing reconstruction on the Cu(100) surface, according to studies of Eren et al. In their other work, they conducted experiments where structural changes of clean and oxygen-covered Cu(100) surfaces under methanol vapor in the 10−200 mTorr pressure range at RT were studied, in light of HP-STM and AP-XPS [69]. As discussed previously, a small amount of atomic oxygen and OH groups are easily adsorbed on the surface of Cu(100), thus introducing the dark spots (depressions in STM contrast) in Figure 7a. When the Cu(100) surface was exposed to CH_3_OH at a pressure of 5 × 10^−8^ Torr, the intensity of dark spots on surface reduced and completely disappeared under CH_3_OH at a pressure of 3 × 10^−6^ Torr (Figure 7b). This is in good agreement with the AP-XPS data: atomic oxygen reacts with methanol to produce methoxy or reacts with methoxy to produce formate or CH_2_O. Other distinguishable dark spots can be observed in Figure 7b, which suggests products diffused rapidly across the surface. Meanwhile, in continuously increasing CH_3_OH pressure to 0.01 Torr, the c (2 × 2) structure could be clearly observed across most of the Cu(100) surface in Figure 7d, except for the lower right region enclosed by a yellow broken line that has not yet been determined. Figure 7e shows an atomically resolved image of Cu(100)-(2$\sqrt{2}\;\times \;\sqrt{2}\;$R45°)-O, as explained above. Surprisingly, the structure remained intact for 0.5 h under up to 0.2 Torr of CH_3_OH, as shown in Figure 7f. This is consistent with AP-XPS data again: no change in atomic oxygen intensity was observed.

The relevant AP-XPS data are shown in the right panel in Figure 7. The main adsorbed methoxy species (marked ξ) is assigned to peaks at around 530.2−530.4 eV of O 1s and 285.2−285.5 eV of C 1s in the binding energy scale. Gas phase methanol (marked β) peaks are observed at 288.0 eV of C 1s and 534.2 eV of O 1s, and formate (marked γ) peaks are located at around 531.4 and 287.5 eV. Authors proposed that adsorbed methanol reacted with the adsorbed O originating from the segregation of O in the subsurface region of the Cu crystal to form methoxy, which in turn formed CH_2_O (marked α), assigned to peaks at ~290 and 534.5 eV. After the introduction of O_2_, a peak assigned to gas phase O_2_ appeared at 539 eV (marked χ). Because no atomic oxygen peak was observed at 529.6 eV, it is considered that atomic oxygen immediately reacts with methoxy to form CH_2_O (290 and 534.5 eV) and formate (287.5 and 531.4 eV). Moreover, no formation of CO2 was observed, indicating that formate was not further oxidized. In order to determine the functional relationship between coverages of different species and pressures, the authors normalized the peak intensities with respect to the Cu 3p peak intensity measured at the same kinetic energy. As a result, the intensity of methoxy increased with methanol pressure up to 0.01 Torr and was subsequently kept constant. After the introduction of O_2_, the intensity of methoxy decreased, while the intensity of formate increased, and the CH_2_O intensity increased with the increasing O_2_ pressure in the gas mixture. It is worth noting that the formate coverage increased with an increasing methanol pressure, which is believed to be due to the oxidation of methoxy to formate by water impurities.

In summary, the following conclusions are given by Eren et al. with the help of HP-STM and AP-XPS. Methanol cannot induce restructuring of the Cu(100) surface but forms a methanol c (2 × 2) overlayer on the Cu(100) surface at RT under pressure up to 0.2 Torr. Adsorption of methanol will be hindered when the Cu(100) surface is pre-adsorbed with a saturated, ordered oxygen layer; thus, the c (2 × 2) structure cannot be observed. Methanol will dissociate and react with oxygen, producing methoxy, formate, and formaldehyde, with the oxygen layer coverage below saturation.

### 3.2. Reactions on Platinum-Based Catalysts

**Figure 8:**
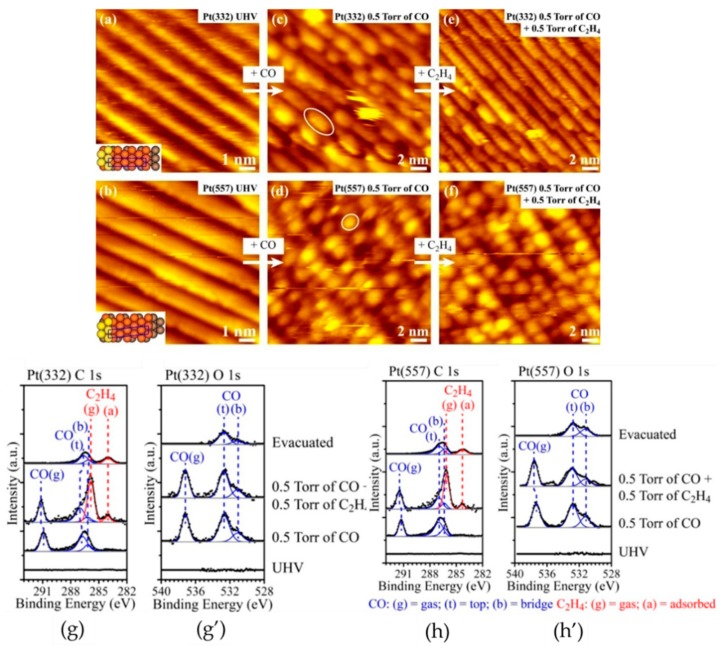
STM images of the Pt(332) surface (**a**) in ultra-high-vacuum (UHV). (**c**) Formation of clusters on Pt(332) in the presence of 0.5 Torr CO. (**e**) Clusters gradually disappear after addition of 0.5 Torr of C_2_H_4_, similar images are shown in (**b**,**d**,**f**) on the Pt(557) surface. Two clusters in each (**c**) and (**d**) are highlighted by a white ellipse as examples. The same AP-XPS experimental data are shown in the right panel. (**g**,**h**) C 1s spectra of Pt(332) and Pt(557) after adding CO and subsequently C_2_H_4_, and (**g’**,**h’**) corresponding O 1s spectra of Pt(332) and Pt(557). From bottom to top: in UHV, under 0.5 Torr of CO, under 0.5 Torr of CO + 0.5 Torr of C_2_H_4_, evacuated to 10^−8^ Torr.

Similar to the case of oxygen pre-adsorption on copper, pre-adsorption of CO or alkynes on different Miller-indices of Pt is also a hot topic because of the outstanding catalytic performance of CO. According to published reports, ethylene adsorption and hydrogenation will be blocked when CO adsorbs first on Pt [70]. Zhu et al. demonstrated how different step geometry sites affects the reconstruction of stepped Pt(332) and Pt(557) under co-adsorption of CO and C_2_H_4_ gas mixtures at room temperature by HP-STM and AP-XPS [71]. Figure 8a,b reveals atomic images of Pt(332) and Pt(557) under UHV. The average widths of visible terraces were ~1.4 nm on both surfaces. A large number of clusters, roughly parallelogram in shape for Pt(332) (Figure 8c) or triangles for Pt(557) (Figure 8d), appeared on both surfaces when 0.5 Torr of CO was introduced at room temperature. Two of the clusters are enclosed by the white ellipses, as shown in Figure 8c,d. Peaks of the CO gas phase adsorbed at the top or bridge sites can also be observed in the corresponding AP-XPS spectra. In the presence of 0.5 Torr of CO, the CO coverage was estimated to be 0.88 ML on Pt(332) and 0.94 ML on Pt(557) according to relevant O 1s and Pt 4f peak areas. The content of CO changed after 0.5 Torr C_2_H_4_ was introduced. The concentration of clusters on Pt(332) distinctly decreased (Figure 8e), while it was unchanged on Pt(557) (Figure 8f). In supporting experiments, more clusters disappeared when the C_2_H_4_ pressure was further raised to 2.5 Torr, leaving the original 570 pm periodic clusters at the step edges. AP-XPS data changed accordingly after the introduction of 0.5 Torr of C_2_H_4_. Extra peaks of the C_2_H_4_ gas phase at ~286.0 eV and a small feature of ethylene adsorbate at ~284.1 eV appeared in the C1s spectra. The coverage of C_2_H_4_ estimated from C1s peak areas was 0.08 ML on Pt(332) and 0.05 ML on Pt(557), and the coverage of CO decreased to 0.80 ML on Pt(332) and 0.88 ML on Pt(557), indicating that C_2_H_4_ can adsorb on the Pt surface that has been pre-adsorbed by CO, by replacing CO molecules.

At first, the reason why the cluster concentration on Pt(332) decreases with a lower CO coverage is because CO–CO repulsion at high CO coverage drives the cluster formation. This consideration is overthrown by contrasting experiments with pure CO on Pt(332) at 7 mTorr (0.73 ML coverage), which induces a higher cluster concentration than that in CO–ethylene mixture (0.8 ML CO coverage). Another possibility for the decrease in cluster concentration is due to the unreconstructed steps not being covered completely by clusters. This judgement is also ruled out by supporting experiments, where differences in step geometry and ethylene adsorption on both surfaces are considered. Upon adsorption of mixed CO and C_2_H_4_, ethylidyne among the products of C_2_H_4_ is the most stable at 3-fold sites on Pt(332), while quad-σ acetylene is the major adsorbate at 4-fold step sites on Pt(557). The adsorption capacity of acetylene is stronger than that of quad-σ acetylene on Pt. The CO poisoning effect of ethylene adsorption on Pt(332) is, thus, inferior to that of Pt(557).

**Figure 9:**
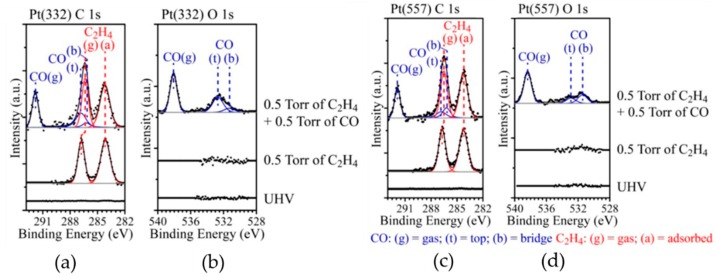
(**a**,**c**) C 1s spectra of Pt(332) and Pt(557) after adding C_2_H_4_ and subsequently CO, and (**b**,**d**) corresponding O 1s spectra of Pt(332) and Pt(557). From bottom to top: in UHV, under 0.5 Torr of C_2_H_4_, under 0.5 Torr of C_2_H_4_ + 0.5 Torr of CO.

Experiments for how different sites of step geometry affect the reconstruction of stepped Pt(332) and Pt(557) are also carried out by reversing the sequence of CO and C_2_H_4_ exposures [72,73]. Extra addition of 0.5 Torr of CO does not give rise to significant cluster changes in STM images of Pt surfaces pre-adsorbed by C_2_H_4_. Figure 9 shows the C 1s and O 1s spectra of Pt(332) and Pt(557) surfaces under 0.5 Torr of C_2_H_4_ and after subsequent addition of 0.5 Torr of CO. In the presence of 0.5 Torr of C_2_H_4_, peaks of the C_2_H_4_ gas phase at ~286.2 eV and a small feature of ethylene adsorbates at ~284.2 eV matched the assignments in Figure 8. After introducing 0.5 Torr of CO, peaks of top-CO sites and bridge-CO sites appeared in the O 1s spectra at ~532.8 and ~531.3 eV, which indicates that CO can be accommodated on stepped Pt surfaces precovered by C_2_H_4_. At the same time, the gas peak of C_2_H_4_ decreased by 0.5 eV, which is due to the increase of sample work function, as evidenced by the red-shift of the C−O stretch in infrared spectroscopy. From the deconvolution of O 1s spectra in Figure 9c,d, it is distinctly noticed that CO mainly occupied top sites on Pt(332) and bridge sites on Pt(557). In conclusion, the influence of the Pt surface with different step geometries is not caused by small differences or the initial coverage of external gases, rather, it is a consequence of the peculiar structural (electronic and chemical) properties of the different step orientations of Pt(332) and Pt(557).

**Figure 10:**
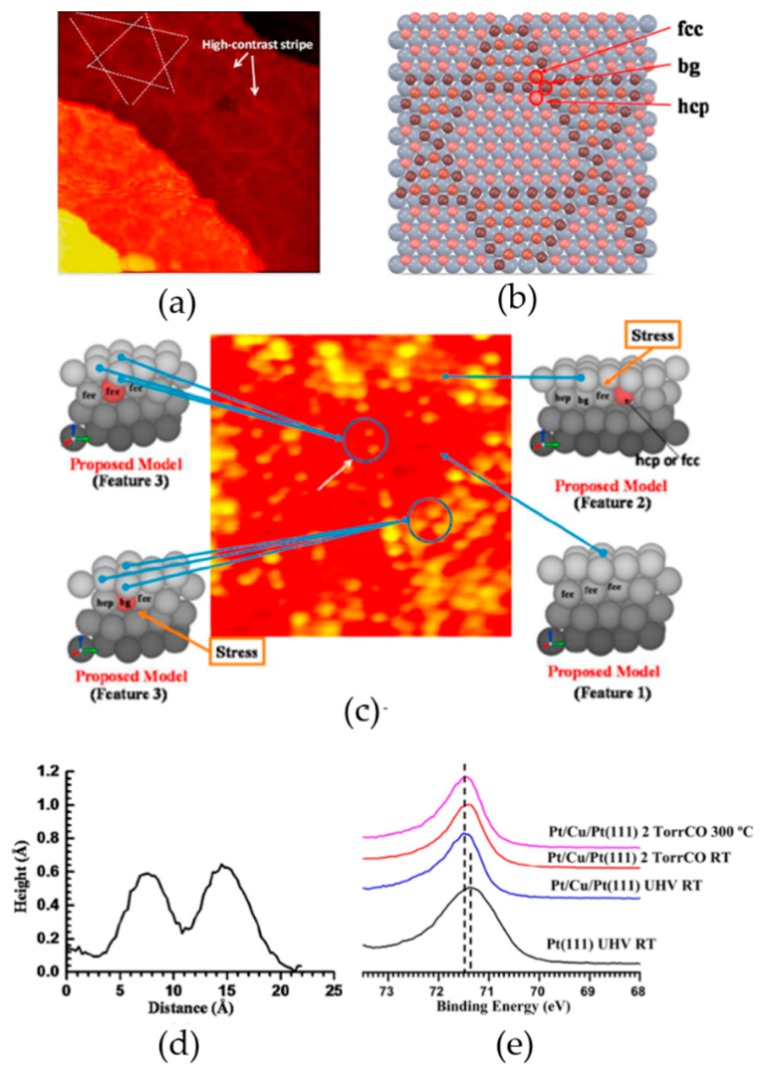
(**a**) STM image of the Pt/Cu/Pt(111) surface under UHV. A basic structure marked by white lines forms high-contrast stripes, which is clearly shown in (**a**). (**b**) Structural model of the Pt/Cu/Pt(111) surface for the formation of stripes. The first layer of atoms is neglected, and dark yellow and brown atoms are the second layer (the alloy layer), in which atoms are either Cu or Pt. Light blue atoms are the third layer of the structure. (**c**) Atom-resolved visualization STM image of Pt/Cu/Pt(111) NSA with a size of 75 × 75 Å at room temperature under UHV. (**d**) Line profile of the section marked with a white arrow in (**c**). (**e**) Pt 4f spectra of Pt(111) and Pt/Cu/Pt(111). From bottom to top: in UHV at RT, in UHV at RT, under 2 Torr of CO at RT, and under 2 Torr of CO at 300 °C.

Besides homogeneous catalysts mentioned above, bimetallic and trimetallic catalysts are also widely applied in industrial applications, and their catalytic mechanisms are widely understood [74,75,76,77,78,79,80]. Compared to homogeneous catalysts, bimetallic and trimetallic catalysts have the advantages of favorable selectivity, better catalytic activity, and reduced deactivation rates. Taking the catalytic capacity of metal atoms for CO as an example, models of Pt/Cu, Pd/Cu, Cu/Pt, and so on, are widely investigated in scientific research because of their simple simulation and preparation. Many groups have studied how such bimetallic catalysts with different coordination environments of bi- and trimetallic crystals influence the CO catalytic oxidation in Torr pressure by HP-STM and AP-XPS in recent years. Zeng et al. detailed three different coordination environments of Cu in a near-surface alloy (NSA) model catalyst Pt/Cu/Pt(111) in UHV and in the presence of 2 Torr CO [81]. After deposition of approximately 1 ML of Cu on Pt(111), highly contrastive, hexagonal-like bright stripes (Figure 10a) under UHV were observed, indicating the formation of Pt/Cu/Pt(111) NSA. It has been defined that three different atom packing types exist: fcc sites (3-fold sites without the bulk atom being underneath), hcp sites (3-fold sites with one bulk atom being underneath), and bridge sites (2-fold sites) (Figure 10b) [82,83]. The stripes are caused by the structure’s transformation from fcc to hcp stacking [84]. These three different features are consistent with three types of Cu atoms with different coordination environments proposed in Figure 10b [84]. Features corresponding to three different coordination environments of Cu in Pt/Cu/Pt(111) NSA are shown by the atomic-resolved STM image in Figure 10c. Feature 1 illustrates the least bright protrusions, which correspond to the Pt atoms on the top layer coordinated by the Pt atoms on the second layer. Feature 2 represents the moderate bright protrusions resulting from the stress induced among atoms stacked in the three different sites mentioned above. Feature 3 represents the brightest protrusions, where Pt atoms packed in hollow sites are coordinated exactly with underneath Cu atoms in the second layer. A complete depression created by a Cu atom coordinating in the top surface layer [84] does not appear in the STM image of Pt/Cu/Pt(111) NSA surface. The authors think it is reasonable that there are no such Cu atoms incorporated in the top surface layer of Pt/Cu/Pt(111) NSA. In addition, the authors carried out in situ experiments of Pt/Cu/Pt(111) and Pt(111) in the presence of 2 Torr CO by HP-STM. Results showed that the top layer of Pt/Cu/Pt(111) was restructured into nanoclusters with an average of 1.6 nm in size, while Pt atoms on the Pt(111) surface would not restructure in CO, as reported before. A promising candidate reason is the interaction between the surface layer Pt and the layer underneath is weakened by Cu insertion because of the size difference between Cu and Pt atoms.

At this point, several comparable experiments were carried out in AP-XPS systems, in which Pt/Cu/Pt(111) was prepared with the same experimental parameters of evaporation as used in the HP-STM system. As shown in Figure 10e, the binding energy of Pt 4f of Pt/Cu/Pt(111) was 0.15 eV higher than that of pure Pt(111). The conclusions can be drawn as follows: the difference was due to the incorporation of Cu atoms of Pt/Cu/Pt(111) with a much smaller diameter than Pt atoms. The interaction of superficial surface layers of Pt/Cu/Pt(111) was weaker than those of pure Pt(111). No significant changes were observed between the binding energy of Pt 4f of Pt/Cu/Pt(111) under 2 Torr CO at RT and a clean Pt(111) in UHV at RT. Compared to Pt 4f of Pt/Cu/Pt(111) in 2 Torr CO at RT, a left-shift of 0.15 eV occurred again for Pt 4f of Pt/Cu/Pt(111) in 2 Torr CO at 573 K (Figure 10e). It can be reasonably speculated that the distribution of Cu atoms changed from UHV to 2 Torr CO environments, which is indeed consistent with the formation of Pt nanoclusters observed in HP-STM. It is proven that Cu atoms are capable of enhancing binding between surface Pt atoms and CO molecules [85] because Cu atoms could migrate to the surface and alloy with Pt atoms when Pt/Cu/Pt(111) NSA is exposed to 2 Torr CO at 573 K, even though it is known that the binding degree of Cu and CO is lower than that of Pt and CO.

**Figure 11:**
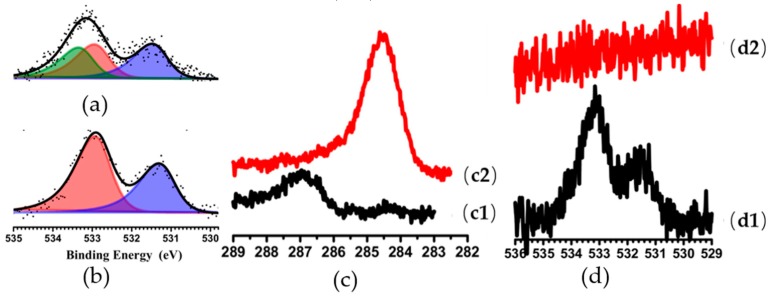
O 1s peaks of CO chemisorbed on pure (**a**) Pt(111) and (**b**) Pt/Cu/Pt(111) in 2 Torr CO at 300 K. C 1s of CO chemisorbed on Pt/Cu/Pt(111) (**c1**) in 2 Torr CO at 300 K and (**c2**) in 2 Torr CO at 573 K. O 1s of CO chemisorbed on Pt/Cu/Pt(111) (**d1**) in 2 Torr CO at 300 K and (**d2**) in 2 Torr CO at 573 K.

In addition, AP-XPS data for several sets of contrastive experiments are shown in Figure 11. The left panel of Figure 11 shows O 1s peaks of Pt(111) and Pt/Cu/Pt(111) in 2 Torr CO at room temperature. In terms of Pt(111), O 1s peaks at 531.3 and 532.9 eV correspond to CO molecules adsorbed on bridge sites and on top sites on Pt(111). O 1s peaks of CO adsorbed on Pt/Cu/Pt(111) have higher binding energies at 531.5 and 533.2 eV. The O1 peaks at 531.5 eV shift by 0.2 eV, and its peak area also decreases at the same time. Similar shifts of binding energy are assigned to CO molecules adsorbed on nanoclusters of Pt atoms, due to restructuring of Pt(557) in 0.5 Torr CO [86]. O 1s peaks at 531.5 and 532.9 eV correspond to CO molecules adsorbed on bridge sites and on top sites of nanoclusters on the surface, respectively. Another new peak at 533.3 eV could be assigned to CO adsorbed at edge of formed nanoclusters. The Figure 11c1,c2,d1,d2 shows C 1s and O 1s peaks of Pt/Cu/Pt(111) in 2 Torr CO at room temperature (black lines) and 573 K (red lines). At room temperature, the binging energy at 287 eV is similar to the chemisorbed CO on the Pt(111) surface [87,88,89]. The corresponding O 1s peak at 533 eV is also detected and shown in Figure 11d1. With the temperature increased to 573K, the original chemisorbed CO peak disappears and is replaced by a new peak at 284.6 eV. No O signal is observed by XPS spectroscopy in this process. It is considered that dissociation of CO molecules on surface of Pt/Cu/Pt(111) occurs at 573 K. Carbon atoms are left to surface and accumulate subsequently. The new peak at 284.6 eV can be assigned to the coke-like carbon adsorbed on the surface of Pt/Cu/Pt(111).

## 4. Conclusions and Perspectives

Taking into account the vital role of surface science in various fundamental research fields, the development of in situ or operando characterization tools with a focus on the surface/interface has become more and more crucial in exploring reactions/materials with atomic/molecular precision under real atmosphere. Encouragingly, successful applications of HP-STM and AP-XPS have been demonstrated in situ from literature reports investigating changes of atomic structures and electronic characteristics towards the study of actual reaction mechanisms on the surface and the identification of structure–property relationships, in which the catalytic reaction with CO involved in various model systems has been the specific focus.

As demonstrated in this review, dynamic atomic structures can be obtained directly under realistic conditions by HP-STM during the reaction process. Meanwhile, changes of core level orbitals in each reactant can also be measured directly from AP-XPS. With such complementary information, detailed mechanisms of catalytic reactions and dynamics of reaction steps can be concluded for various surfaces or interfaces with the inclusion of DFT calculations, such as the location of bond breaking, adsorption sites, and the active center. We have discussed in the main text that the combination of HP-STM and AP-XPS tools are proven to be a powerful and efficient approach to explore catalytic mechanisms based on model catalysts such as transition metal and noble metal surfaces. 

Despite the great achievements of HP-STM and AP-XPS, there are still some intrinsic limitations. First, samples are strictly limited to ideal surfaces with atomic roughness for HP-STM measurements. AP-XPS does not ask for an atomically smooth surface, but still, a relatively flat surface needs to be prepared before measurement. This requirement excludes lots of samples that are just catalysts in real reactions, for example, powders, clusters, rods, noncrystallized surfaces, and so on. To overcome this bottleneck, some other tools need to be introduced in combination with AP-XPS and HP-STM. For example, in situ X-ray absorption spectroscopy (XAS), X-ray Raman spectroscopy (XRS), and in situ transmission electron microscopy (TEM) can be useful supplementary measures. Secondly, because of the high sensitivity of photoelectrons to scattering, electron detection still has to be performed under UHV, which definitely restricts the high-pressure limit in the reaction cell/chamber, even though differential pumping systems are introduced. In fact, real catalytic reactions might need several hundred bars to trigger some reactions, and the upper limit of 100 mbar at present in AP-XPS is, thus, far away from industry applications. Therefore, in situ XAS measurements that can hold high-pressure reaction cells will be importantly helpful. Thirdly, with the photoenergy up to 2000 eV at present for AP-XPS measurements, deep interfacial or bulk structures cannot be observed because of the limited escape depth of photoelectrons of about a few nanometers, whilst STM can only detect the electronic states from the atoms of the first layer or second layer that are mostly on the surface. Based on this, a wide photoenergy range beamline for AP-XPS is crucially necessary, which can investigate both surface layers and deep interfacial layers by tuning the kinetic energy of photoelectrons and, therefore, the detection depth. Meanwhile, in situ TEM can also be a useful method to get on-demand information from deep layers inside the bulk by measuring the cross-sections. Nevertheless, the combination of AP-XPS and HP-STM will continue to be a feasible and efficient measure in the field of surface/interface catalysis exploitation, while more insights will be obtained once united with other characterization tools.
